# Social Disparities in Health Literacy in the United States

**DOI:** 10.3928/24748307-20190131-01

**Published:** 2019-03-08

**Authors:** Sasha A. Fleary, Reynolette Ettienne

## Abstract

Health literacy has been identified as a contributor to both health disparities and social determinants of health. There is significant overlap in demographic characteristics of those who are at risk for health disparities and low health literacy. This study expanded the research on health literacy and disparities by quantifying the social disparities within health literacy using a relatively new methodology. We analyzed data from the 2013 Health Information National Trends Survey (*N* = 1,675). The Extended Gastwirth Index was used to determine the disparities in health literacy. Participants who were female, age 18 to 34 years, White, highly educated (postbaccalaureate), and with incomes ≥$200,000 had the highest health literacy and served as the reference groups. Males, age 35 to 49 years, who had high school graduates, and people who were Hispanic and with incomes between $50,000 and $74,999 had the highest disparities in health literacy. Income (∼30%) and education (∼37%) were the highest contributors to overall disparities in health literacy, whereas sex (∼3%) was the lowest contributor. The overwhelming contribution of income and education to disparities in health literacy and the range of disparities within these demographic characteristics highlight populations that could benefit from tailored interventions to improve their health literacy. **[*HLRP: Health Literacy Research and Practice*. 2019;3(1):e47–e52.]**

Health disparities and low health literacy (HL) remain significant public health challenges (Meyer, Yoon, & Kaufmann, 2013). Many Americans are unable to comprehend or act upon health information and have basic or below-basic HL (Kindig, Panzer, & Nielsen-Bohlman, 2004). HL is conceptualized as both a social determinant of health (SDH) (Sentell, Baker, Onaka, & Braun, 2011) and a tool to empower people to exercise control over modifiable SDH (Nutbeam, 2008; Rowlands, Shaw, Jaswal, Smith, & Harpham, 2015). Further, HL is necessary to develop and maintain health as it influences prevention services utilization, medical adherence, disease status satisfaction, and health decision-making involvement (Baker et al., 2002; Mancuso & Rincon, 2006; Scott, Gazmararian, Williams, & Baker, 2002).

Populations most at risk for health disparities in chronic diseases and low HL are similar (Bennett, Chen, Soroui, & White, 2009; Kutner, Greenburg, & Paulsen, 2006; Paasche-Orlow & Wolf, 2010). People who are older (Cutilli, 2007), racial and ethnic minorities (Kutner et al., 2006), non-native English speakers, and with low income and/or education are at greater risk for low HL (Kindig et al., 2004; Sudore et al., 2006). Most of the cited studies determined the relationship between HL and sociodemographic characteristics rather than quantifying the differences in HL between groups. Quantifying disparities in HL has the added benefit of informing needs-based resource allocation necessary for improving population HL and reducing disparities in HL.

Using national HL data, the goal of the current study was to use the Extended Gastwirth Index (Asada, Yoshida, & Whipp, 2013) to quantify the disparities within HL using population characteristics indicative of health disparities (education, income, age, race, sex). This noncorrelational methodology, the Extended Gastwirth Index, uses between-group comparisons to calculate a disparities ratio for each demographic characteristic and calculates an overall disparities ratio. In quantifying the person and collective contributions of sociodemographic characteristics in health disparities, this methodology provides valid indices/percentages for informing targeted intervention efforts and comparing changes in disparities over time.

## Methods

### Participants

We analyzed data from the 2013 U.S. Health Information National Trends Survey (HINTS) 4 Cycle 3 (National Institutes of Health, 2013), administered by the National Cancer Institute. HINTS employs a complex probability sampling design, collecting data from noninstitutionalized civilian adults. Participants who answered all four HL questions were included in our analyses (*N* = 1,675). More information about the survey methodology is available on the HINTS website. As HINTS is a de-identified publicly available dataset, no further Institutional Review Board approval was needed for this research.

### Measures

*Sociodemographic variables*. Participants self-reported sex, age, household annual income, and education. Participants' self-identified ethnicity and race, and responses were combined into Hispanic, non-Hispanic White, non-Hispanic Black, and non-Hispanic other (American Indian or Alaska Native, Asian, Native Hawaiian or Pacific Islander).

*Health literacy*. HL was measured using a modified, self-administered form of the Newest Vital Sign (NVS) (Weiss et al., 2005). Four questions based on an ice cream nutrition label assessed numeracy and reading skills. Questions five and six from the original NVS were not included as skip logic procedures were needed. Responses were scored and dichotomized into *high* (4 correct) and *low* (<4 correct) HL. These cutoff scores are consistent with the scoring on the full measure.

### Statistical Analysis

Descriptive analyses were computed using proc survey-freq and proc surveymeans commands in SAS 9.4 to allow for weighting the data to reflect United States population demographics. Both jackknife replicate weights and final sample weights were used. Microsoft Excel spreadsheets were used to calculate disparity ratios. Overall, disparities and sociodemographic characteristic-specific disparities in HL were calculated using the Extended Gastwirth Index. This method, which summarizes social disparities in health, has several advantages including sensitivity to group size, allowance for between-group comparisons within a characteristic, and calculation of a summary ratio of disparities across characteristics. Ratios were computed as follows: (1) Determine the reference group: extract fraction share for subpopulations (% with high HL) within each characteristic. The reference group has the highest fraction share; (2) Calculate within characteristic-specific disparity: [Reference group fraction share minus other group fraction share] X other group population share; (3) Calculate characteristic-specific disparity: sum of group disparities within characteristic; (4) Overall disparities: average of all characteristic-specific disparities; (5) Characteristic contribution to overall disparities: [characteristic-specific disparities ratio/sum of all characteristic-specific ratios] × 100.

**Figure 1.**
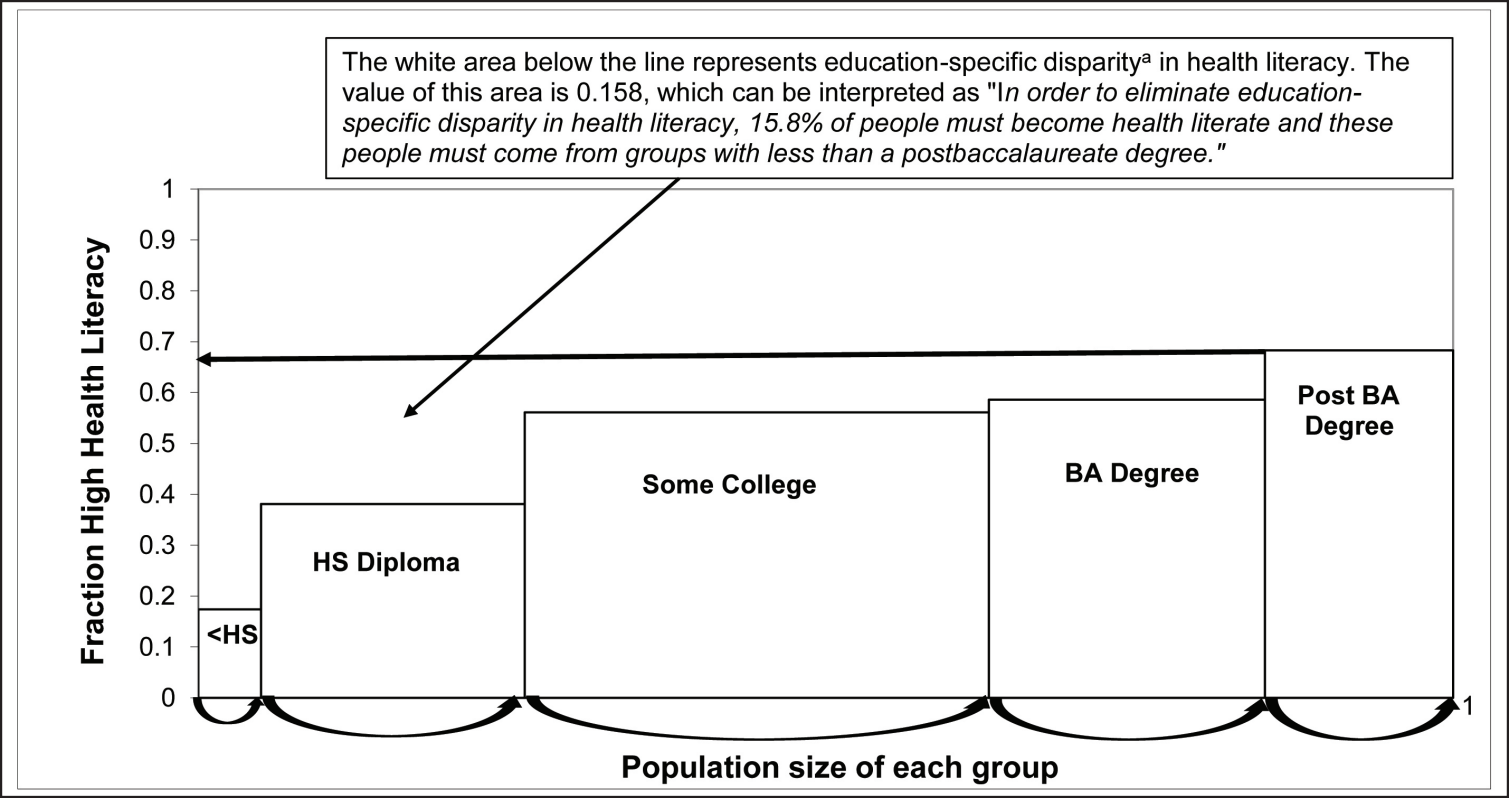
A visual of education specific disparity in health literacy. ^a^Education-specific disparity (calculation) = ([Reference group fraction with high health literacy {Post BA degree} – Group 1 fraction with high health literacy {<HS}] × Group 1 population share) + ([Reference group fraction with high health literacy {Post BA degree} – Group 2 fraction with high health literacy {HS diploma}] × Group 2 population share) + ([Reference group fraction with high health literacy {Post BA degree} – Group 2 fraction with high health literacy {Some college}] × Group 3 population share) + ([Reference group fraction with high health literacy {Post BA degree} – Group 4 fraction with high health literacy {BA degree}] × Group 4 population share). Education-specific disparity in health literacy = ([.683 − .174] × .058) + ([.683 − .381] × .204) + ([.683 − .561] × .369) + ([.683 − .586] × .228) = .158. HS = high school; BA = baccalaureate.

**Figure 1** illustrates the calculation of education-specific disparities ratios. Disparity ratios range from zero to one with higher ratios representing higher disparities between groups. Within group-specific disparity ratios were imputed into SPSS to match respondents' demographic characteristics. One sample *t* test determined if the mean disparity ratio was significantly different from zero.

## Results

More than one-half of the participants were White, female, had household incomes ≥$50,000, and had at least some college education. Groups with a majority of people with high HL include females, age 18 to 64 years, White, non-Hispanic other (American Indian or Alaska Native, Asian, Native Hawaiian or Pacific Islander), those with at least some college education, and incomes ≥$35,000. There were significant group differences for all characteristics except sex. Sociodemographic characteristics, disparities ratios, and *t* statistics are presented in **Table 1**.

The *t* tests were significant suggesting that the mean of the characteristic-specific disparities ratios differed significantly from zero. Overall HL disparity was 0.0853, suggesting that to eliminate HL disparities 8.53% of adults from nonreference groups need to improve HL. Income (∼30%) and education (∼37%) were the highest contributors to overall disparities in HL, whereas sex (∼3%) provided the lowest contribution. Age and race contributed similarly (∼15%) to overall disparities in HL.

Females were the reference group for sex. Sex-specific disparities were 0.0140, suggesting that 1.4% of males needed to improve HL to eliminate disparities. Participants age 35 to 49 years had the highest age-specific disparities when compared to the reference group (age 18 to 34 years). The age-specific disparity ratio was 0.0621, suggesting that 6.21% of people from nonreference groups need to improve HL to eliminate age-specific disparities. Non-Hispanic White participants were the reference group, whereas Hispanic participants had the highest race-specific disparities. Race-specific disparity was 0.0624, suggesting that 6.24% of non-White people need to improve HL to eliminate race-specific disparities. Participants with a high school diploma had the highest education-specific disparities compared to the reference group (postbaccalaureate). Education-specific disparity was 0.1583, suggesting that 15.83% of people who did not have a postbaccalaureate education need to increase HL to eliminate education-specific disparities. Participants with incomes between $50,000 and $74,999 had the highest income-specific disparities compared to the reference group (≥$200,000). Income-specific disparity was 0.1295, suggesting that 12.95% of people with household incomes <$200,000 need to improve HL to eliminate income-specific disparities.

## Discussion

This research quantified social disparities in HL using the Extended Gastwirth Index, which allows for quantifying the individual and collective contributions of sociodemographic characteristics in health disparities. We can conclude from **Table 1** that to eliminate disparities in HL, we need to improve the HL of ∼3% of males, ∼6% adults ≥35-years-old, ∼6% racial/ethnic minorities, ∼13% of people with incomes <$200,000, and ∼16% of people who do not have a postbaccalaureate education. One HL intervention cannot address the needs of all groups. Instead, interventionists may choose to target the most disparate groups within the characteristics with the highest contributors to HL disparities (e.g., high school graduates within education).

The results of this study support continued work on improving HL in communities with lower income and lower education. People who have lower income/education rates are more likely to have public health insurance (Majerol, Newkirk, & Garfield, 2014), poorer access to health resources (Kushel, Gupta, Gee, & Haas, 2006), and stressors that make practicing HL difficult (McCray, 2005). Intervention strategies for this group should be considerate of these barriers when providing skills for accessing and using health information and interacting with the health system.

Research on HL and sex is emerging. Some studies found that women had higher HL than men (Kutner et al., 2006; Von Wagner, Knight, Steptoe, & Wardle, 2007), although others found no sex differences (Paasche-Orlow & Wolf, 2010). Our results support the latter, as sex-specific disparity in HL contributed less than 4% to overall disparities.

The reference groups for race and age were White and people age 18 to 34 years, respectively. Both characteristics contributed ∼15% to the overall disparities ratio, which is almost one-half of the contributions observed for education and income. Given the relationship between race, education, and income (Braveman et al., 2011), designing and implementing interventions to address income and education disparities may indirectly address some of the race-specific disparities in HL.

Strengths of our study include the use of a novel methodology. The Extended Gastwirth Index shifts measuring health disparities from a single characteristic to assessing group disparities specific to characteristics. Previous disparities research used regression methods (Scott et al., 2002) for measuring health disparities; however, these correlational approaches limit the use of the results to make programmatic decisions. Specifically, correlational approaches may highlight sources of disparities but do not provide sufficient data to inform resource allocation for addressing the group with the highest disparities or for assessing shift in sources of disparities over time as is possible using the Extended Gastwirth Index. Further, correlational approaches may not account for the size of the group in question, or whether the reference group (usually the “healthiest”) can serve as a reference for all characteristics being assessed (Asada et al., 2013). The usability of the Extended Gastwirth index ratios for identifying and designing interventions for people at-risk is also a strength.

## Study Limitations

Conversely, the research is not without limitations, as the Extended Gastwirth Index does not account for the additive or multiplicative burden of belonging to more than one disparities risk group. As well, the NVS measures functional HL (Weiss et al., 2005); thus, we cannot generalize our findings to other types of HL. A truncated version of the NVS was used in this study; however, the original threshold for high HL was maintained. It is possible that people may have met the high HL threshold if given the full version of the NVS, thus changing the disparities estimates. Race and ethnicity were collapsed due to small sample sizes for minority groups, but ignoring the variation within smaller groups may undermine intervention strategies. Further, the small sample size for people with less than a high school education likely underestimated the group's disparity ratio. Future research should explore disparities within racial groups, recruit sufficient samples within groups, and assess all aspects of HL.

## Conclusions

This study confirms that subgroups most at risk for health disparities are at risk for HL disparities. HL may be a tool to reduce health disparities; however, the disparities within HL must be addressed to make it a useful tool. Further, HL is one of the few changeable contributors to health disparities where individual-level interventions may have a strong effect. Our results are a starting point for determining targetable groups for individual-level interventions to improve HL and reduce health disparities.

## Figures and Tables

**Table 1:** Sociodemographic Characteristics of the Sample and Disparities in Health Literacy (*N* = 1, 675)

**Characteristic**	***n* (%)**	**Participants with All NVS Items Correct (% within the Group)**	**Disparity Ratio^a^**	***M* ± *SE* of Disparity Ratio**	**% Contribution^b^**	***t*/*df*^c^**

Sex		783 (52.8)	0.0140	.0076 ± .0002	3.29	36.43/50
Female	877 (46)	448 (54.2)	0.0140			
Male	679 (54)	335 (51.6)	0			
Missing	119					

Age (years)		829 (52.5)^*^	0.0621	.0138 ± .0003	14.57	49.12/50
18–34	248 (27.3)	147 (58.7)	0			
35–49	418 (32.6)	211 (50.6)	0.0264			
50–64	604 (26.4)	306 (53.2)	0.0145			
65–74	255 (8.1)	124 (51.7)	0.0057			
75+	130 (5.7)	41 (31)	0.0155			
Missing	20					

Race/ethnicity		785 (53.3)^**^	0.0624	.00700 ± .0004	14.64	18/50
Non-Hispanic White	954 (71.8)	576 (59.5)	0			
Hispanic	248 (13.5)	81 (30.9)	0.0386			
Non-Hispanic Black	206 (7.6)	63 (31.7)	0.0211			
Non-Hispanic other	124 (7.2)	65 (55.8)	0.0027			
Missing	143					

Education		833 (52.5)^**^	0.1583	.0359 ± .0006	37.12	61.57/50
<High school	89 (5.8)	17 (17.4)	0.0295			
High school graduate	300 (20.4)	95 (38.1)	0.0616			
Some college	530 (36.9)	260 (56.1)	0.0450			
Bachelor's degree	454 (22.8)	261 (58.6)	0.0221			
Postbaccalaureate	287 (14.1)	200 (68.3)	0			
Missing	15					

Income		817 (52.7)^**^	0.1295	.0145 ± .0003	30.37	45.40/50
$0–$9,999	109 (4.9)	17 (21.8)	0.0215			
$10,000–$14,999	93 (4.6)	21 (27.6)	0.0175			
$15,000–$19999	100 (5.3)	35 (36.5)	0.0154			
$20,000–$34,999	220 (13.9)	91 (47.5)	0.0252			
$35,000–$49,999	237 (14.6)	123 (60.5)	0.0074			
$50,000–$74,999	290 (17.6)	167 (50.5)	0.0266			
$75,000–$99,999	236 (15.5)	136 (57.7)	0.0122			
$100,000–$199,999	248 (16.8)	161 (63.4)	0.0037			
≥$200,000	94 (6.8)	66 (65.6)	0			
Missing	48					

Note.

a Characteristic-specific disparity ratio = [Reference group fraction with high health literacy – Group 1 fraction with higher health literacy] × Group 1 population share + [Reference group fraction with high health literacy – Group 2 fraction with higher health literacy] × Group 2 population share (e.g., high school graduate disparity in health literacy = [Postbaccalaureate fraction share – high school graduate fraction share] × high school graduate population share. Education-specific disparity in health literacy = less than high school disparity ratio + high school graduate disparity ratio + some college disparity ratio + bachelor's degree disparity ratio + postbaccalaureate disparities ratio.

b Percentage contribution = [attribute-specific ratio/sum of all attribute-specific ratio] × 100.

c All *t* statistics were significant at *p* ≤ .001. NVS = Newest Vital Sign; *SE* = standard error.

* *p* < .05.

** *p* ≤ .001.
